# Severe Gastric Ulcerations With Impending Necrosis in a Patient Who Had Gastric Balloon Insertion Following Previous Sleeve Gastrectomy

**DOI:** 10.7759/cureus.22983

**Published:** 2022-03-09

**Authors:** Salim Al Harthy, Abdullah Al Lawati, Meetham Al Lawati

**Affiliations:** 1 Gastroenterology, Royal Hospital, Muscat, OMN; 2 College of Medicine and Health Sciences, Sultan Qaboos University, Muscat, OMN

**Keywords:** endoscopic management of obesity, stomach ulcer, sleeve gastrectomy, endoscopic intragastric balloon, sloughing mucosa, tight peptic stricture

## Abstract

In this report, we discuss the case of a 44-year-old obese female patient who had her recently installed intragastric balloon removed due to ulceration in the gastric mucosa, which would have led to necrosis as shown by oesophago-gastro-duodenoscopy (OGD). In addition, she had symptoms of nausea, vomiting, dysuria, fever, and experienced severe dehydration, which could have resulted in the formation of ureteric and renal stones. Thus, she was rehydrated and was started on antibiotics. She also underwent successful removal of the intragastric balloon aimed at preserving and healing of the remaining gastric mucosa. Post-op findings were unremarkable; however, a tight peptic stricture at the proximal stomach was formed four weeks after her balloon removal.

## Introduction

The obesity rates in Oman have constantly been increasing, with an estimated 30% of the population having a BMI of more than 30 and thus belonging to the overweight/obese categories [1]. This increase has translated into increased demand for both invasive as well as non-invasive approaches to tackle the obesity problem. In terms of the invasive approach, bariatric surgeries, which are a group of surgeries aimed to reduce the patient's weight, have gained prominence in recent years [2]. As for non-invasive approaches, the insertion of an intragastric balloon, which is aimed at reducing food intake has recently gained attention [3]. Both approaches are generally safe but may also have complications. In this report, we present an unusual case of a patient who underwent a sleeve gastrectomy surgery in the past but years later started to gain weight and hence was labeled obese once again. Thus, she had an intragastric balloon inserted to reduce her weight; however, she could not tolerate it and, therefore, her balloon was removed to avoid complications of perforation and necrosis.

## Case presentation

**Figure 1:**
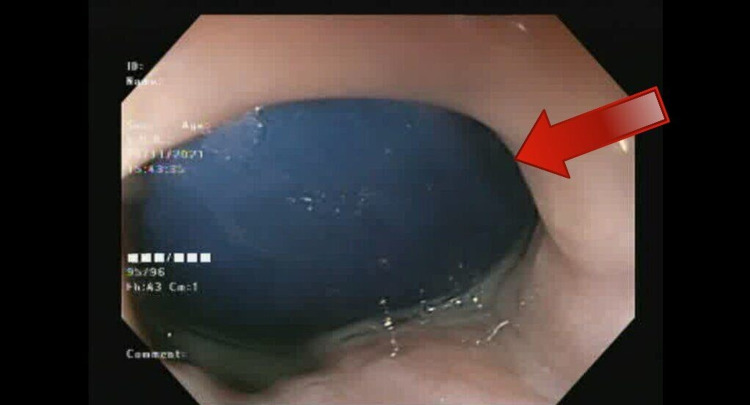
Weight reduction balloon stuck in the proximal portion of the stomach in post gastric sleeve patient.

**Figure 2:**
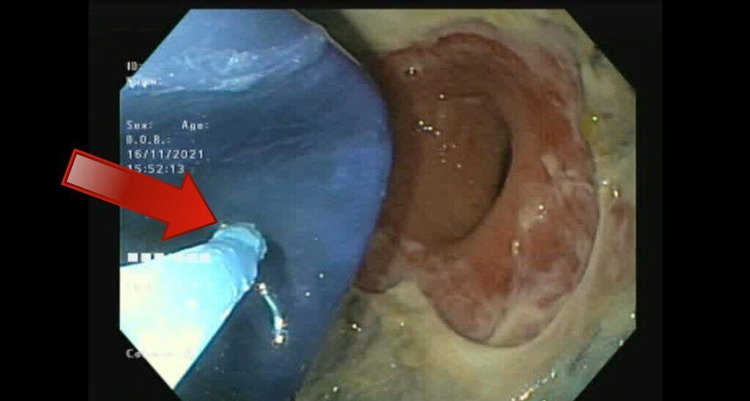
Gastric balloon being aspirated prior to its removal, revealing extensive gastric ulceration due to the pressure effect of the balloon on the mucosa.

**Figure 3:**
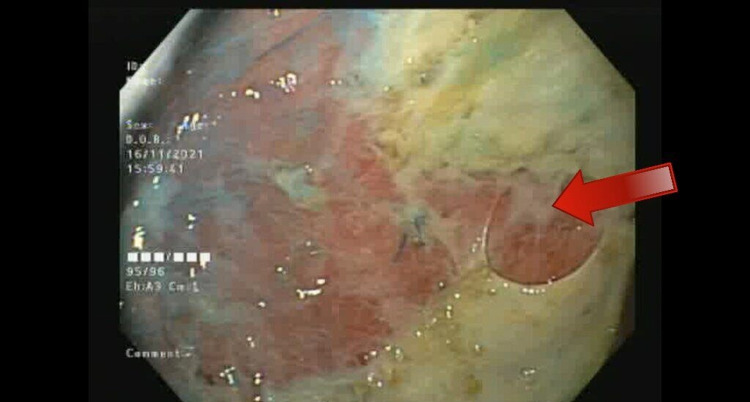
Extensive ischemic pressure related to ulceration of the gastric mucosa after removal of the balloon.

A 44-year-old female patient with a surgical history of laparoscopic sleeve gastrectomy 11 years back, was referred to the emergency department at our tertiary hospital with complaints of recurrent nausea, vomiting, and poor oral intake. She also complained of dysuria, watery diarrhea, and fever. The patient stated that she underwent an intragastric balloon insertion in a foreign country eight days before visiting our hospital. The balloon was ORBERA® (Apollo Endosurgery, Inc.Austin, Texas, United States), and 500ml of normal saline with methylene blue was used to insufflate it. On examination, she appeared to be alert and was not in any form of distress. She had no organomegaly, and her vitals were all normal. However, she did have tenderness in the epigastric area and had right flank pain. Laboratory findings showed elevations in the inflammatory C-reactive protein (CRP) marker value at 306 mg/L value, as well as elevations in the WBC (14*109/L) along with neutrophils (10.7*109/L). Radiologically, the patient was diagnosed as having a ureteric stone and her septic picture was attributed to urinary tract infection (UTI). This was due to the dehydration and extensive stomach ulceration, which may have caused bacteremia resulting in UTI. She was started on a full dose of the broad-spectrum antibiotic piperacillin, tazobactam, and was rehydrated. An oesophago-gastro-duodenoscopy (OGD) was done and showed the gastric mucosa to be ulcerated at the balloon site with much sloughing at the proximal stomach. Thus, the patient was advised to remove her intragastric balloon due to fears of imminent necrosis, to which she agreed. She underwent a successful intragastric removal three days later. Post-operative findings were unremarkable. Figure 1, Figure 2, and Figure 3 show the steps in the intragastric balloon removal procedure.

Four weeks after the intervention, the patient presented with further complaints of nausea, vomiting, and poor oral intake. An endoscopy revealed a severe tight peptic structure at the proximal stomach. This stricture was attributed to the healing process with fibrosis that was caused by the extensive gastric ulceration and necrosis caused by the balloon.

## Discussion

Weight reducing procedures have been divided into invasive and non-invasive procedures. Invasive bariatric surgeries, including the laparoscopic sleeve gastrectomy, vertical banded gastroplasty, and the Roux-en-Y gastric bypass, are the most common procedures [4]. It is estimated that 580,000 people worldwide undergo such surgeries every year [5]. Literature generally shows that invasive bariatric surgeries are generally safe, with one study observing no mortalities and estimating that only 5.1% of the patients experienced complications [6]. Late complications include anastomotic leaks, thromboembolisms, stricture formations, and ulceration [7]. Bariatric surgeries are generally successful in reducing weight, with a study concluding that 95.1% of patients experienced some form of weight loss within the first year of surgery [6]. Being a safe procedure does not eliminate the complications related to major surgeries, especially GI-related surgeries and, thus, this is one drawback. 

As for non-invasive approaches to weight loss, diet and lifestyle modification are the first lines of approach. Following that would be the insertion of an intragastric balloon, which is a temporary method, usually for six months, in which an empty silicon rubber is inserted through the mouth into the stomach and is then filled with saline to expand [8]. This expanded balloon prevents the patient from eating food and induces early satiety by altering the various gut hormones such as leptin, ghrelin, and cholecystokinin [9]. The advantage of the intragastric balloon is that it is temporary and non-invasive and has fewer complications than the surgical approach. On the other hand, the balloon is temporary so the patient is more likely to gain weight following its removal. About 4-7% of patients are forced to remove the balloon due to symptoms of nausea, reflux, and abdominal discomfort [10]. Complications such as perforation due to ulceration or pressure necrosis may also incur but are rare [10].

Given that the surgical approach results in a bigger reduction in weight [11], it is generally the most preferred despite its complications. The non-invasive intragastric approach is a newer approach and is seen as an alternative for those that cannot tolerate the surgical approach for any reason or those who have regained weight after undergoing weight-loss surgeries and thus need a viable alternative, as many complications arise if the bariatric surgeries are done more than once [12]. However, as seen in our case, the patient presented with severe symptoms and complications after undergoing an intragastric balloon insertion given her history of undergoing sleeve gastrectomy surgery. In a study by Genco et al., the overall complication rate of an intragastric balloon following previous sleeve gastrectomy was found to be 2.8% (70/2515 patients) [13]. Further studies are needed to address this issue and to determine whether intragastric balloons are contraindicated in patients with a bariatric surgical history.

## Conclusions

Intragastric balloons are a non-surgical, non-invasive approach to weight reduction. The procedure is reversible and is associated with fewer symptoms and complications compared to the surgical approach. Despite this, intragastric balloons are less utilized as they result in a lower weight reduction than the surgical approach. Intragastric balloons are generally used for patients who cannot tolerate surgery or have regained weight post-surgery; however, as discussed in this case, they may result in symptoms and complications that could lead to perforation and necrosis and, therefore, may not be viable to all patients.
